# Mechanical Control of Organ Size in the Development of the Drosophila Wing Disc

**DOI:** 10.1371/journal.pone.0076171

**Published:** 2013-10-25

**Authors:** Thomas Schluck, Ulrike Nienhaus, Tinri Aegerter-Wilmsen, Christof M. Aegerter

**Affiliations:** 1 Physik-Institut, University of Zurich, Zurich, Switzerland; 2 Institute of Molecular Life Sciences, University of Zurich, Zurich, Switzerland; Centrum Wiskunde & Informatica (CWI) & Netherlands Institute for Systems Biology, The Netherlands

## Abstract

Control of cessation of growth in developing organs has recently been proposed to be influenced by mechanical forces acting on the tissue due to its growth. In particular, it was proposed that stretching of the tissue leads to an increase in cell proliferation. Using the model system of the Drosophila wing imaginal disc, we directly stretch the tissue finding a significant increase in cell proliferation, thus confirming this hypothesis. In addition, we characterize the growth over the entire growth period of the wing disc finding a correlation between the apical cell area and cell proliferation rate.

PACS numbers: 87.19.lx, 87.18.Nq, 87.80.Ek, 87.17.Ee, 87.85.Xd

## Introduction

Control of organ size and growth is a fundamental open question in developmental biology [1]. While a wealth of knowledge on biochemical pathways and their genetic control has been accumulated over the last decades, several issues in organ growth, in particular the question of the cessation of growth, remain unanswered. A widely used model system for the study of organ growth control is the wing imaginal disc of Drosophila [2], which is the larval precursor organ that becomes the wing of the adult fly during metamorphosis. The wing imaginal disc is a two dimensional epithelial tissue which originates from a sac of about 30 cells in the embryo, growing to contain roughly 30 000 cells by the end of larval development. The end-point of growth appears to be autonomous to the disc, since dissected discs which were transplanted into the abdomens of adult flies stopped growing upon reaching the same size as non-dissected discs [3]. While growth can be influenced both by an increase in cell size and an increase in cell number, it is important to note that in wild type wing discs, growth almost exclusively takes place via cell proliferation [1], which is why we will study cell division rates as a measure for growth in the following.

The wing disc is patterned by proteins known as morphogens which have been shown to have a profound influence on growth via different biochemical pathways, thus acting as growth factors [4], [5]. However, there is no direct connection between the concentration of these growth factors and cell proliferation since proliferation occurs roughly uniformly over the entire tissue [6], [7] whereas the morphogens are present in spatial gradients [8], [9]. A solution to this paradox has been proposed in a controlling role for mechanical forces in addition to established molecular growth factors [10]–[13]. In these models mechanical tension has a growth promoting effect and, correspondingly, mechanical compression inhibits growth. Proliferation inside the tissue leads to a specific distribution of mechanical stresses with high compression in the center of the disc, where growth factors are most prominent and tension in the surrounding tissue.

The occurrence of such stresses has been inferred experimentally from birefringence measurements [14], as well as from a characterization of cell-cell interactions based on the proposition that local force balances yield the geometry of the cell shapes in the tissue [15]. Starting from experimental images of cell shapes, [15] solved the inverse problem of force balance, thus determining the local forces and showing that the compressional stress strongly and negatively correlates with the apical area of a cell [15]. In addition, the local strain tensor has been determined for the tissue. It also shows radial tension in the periphery and compression in the center [13].

According to the aforementioned models, stresses lead to a change in the proliferation rates relative to those expected purely from growth factor concentrations. They thus ensure the occurrence of spatially uniform proliferation, as well as regulating the cessation thereof. While it has long been known that division rates in mammalian cell cultures increase upon the application of mechanical tension [16], [17] and also that the growth of cancerous tumors can be inhibited by mechanical compression [18], a regulatory role for mechanical forces has yet to be shown in epithelial tissues, such as those of Drosophila, where a combination of mechano-regulation with known growth-regulatory pathways is feasible [13]. Despite recent advances in relating mechano-regulation in mammalian tissue cultures to biochemical pathways known to be at work in the wing disc [19], the evidence remains indirect coming from cell cultures rather than developing tissues.

Here we will test the basic assumption of the mechanical feedback models that mechanical tension leads to increased proliferation experimentally. This will be done directly by stretching wing imaginal discs with a given force and simultaneously determining the proliferation rate for late third instar wing discs. In addition we characterize the average proliferation over the whole growth period and use the apical cell area as a measure of mechanical strain.

## Experiments

### A. Direct mechanical stretching

**Figure 1:**
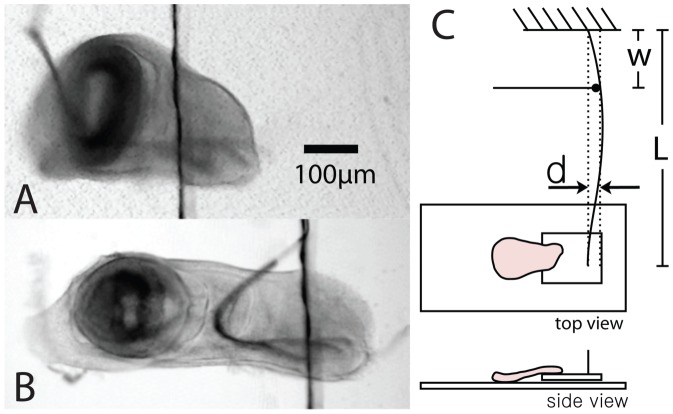
Schematic of the setup. A: Transmission microscope image of a cultured wing imaginal disc in the mechanical stretching setup in the absence of an applied force. B: The same disc after application of a force of 350 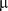
N, leading to a strain of 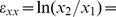
0.19, where 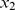 and 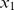 are distances in the stretched and unstretched tissue respectively. Note that the folds in the wing disc present between the wing pouch and the notum have been stretched, leading to a larger stretching in this part of the tissue. C: A schematic illustration of the setup, where the spring sheet of length 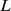 is pushed a distance 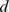 at position 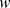 giving rise to a force on the wing disc of 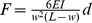. Here, 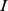 is the area moment of inertia of the spring sheet and 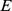 its elastic modulus.

For this purpose, we use in-vitro experiments in which we mechanically stretch discs, which were dissected in PBS and cultured in Clone8 medium [20], using a forcing apparatus described in detail elsewhere [21]. This enables us to apply forces on the order of 10–1000 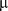
N uniaxially (see Fig. 1). In order to be able to determine the proliferation rate while stretching the tissue, the setup was custom built on an inverted confocal microscope. The overall mechanical response of the tissue is recorded in transmission mode at a magnification of 10×. For the determination of cell outlines, a magnification of 40× is used in fluorescent confocal mode. In both of these modes, the tissue strain can be determined in the direction of the applied forces as well as perpendicular to it. Due to the large strains in the tissue, we use the true strain, 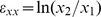, where 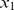 and 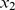 are the distances between easily identifiable mitotic cells in the images taken immediately before and after the application of the force, respectively. Applying the same method in the perpendicular direction, we find a Poisson-ratio 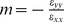. We find that the mechanical strain incurred under uniaxial tension consists of a tensional strain along the direction of the applied force and a compressional strain perpendicular to this direction. This is exemplified by the finite values of the Poisson-ratio of 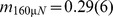 and 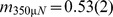, where the number in the parentheses denotes the uncertainty in the last digit of the quoted value.

A transmission image of a wing disc before and after stretching, as well as a schematic drawing of the setup are shown in Fig. 1. The stretching of the tissue which ensues from our application of an external force can be compared with the stretching of the tissue found in-vivo under normal growth conditions during the late second instar stage [22]. There, wing discs were studied in-vivo and dissected immediately afterwards comparing the cell outlines in both cases. The strains observed in this way are of the order of 0.2–0.5.

**Figure 2:**
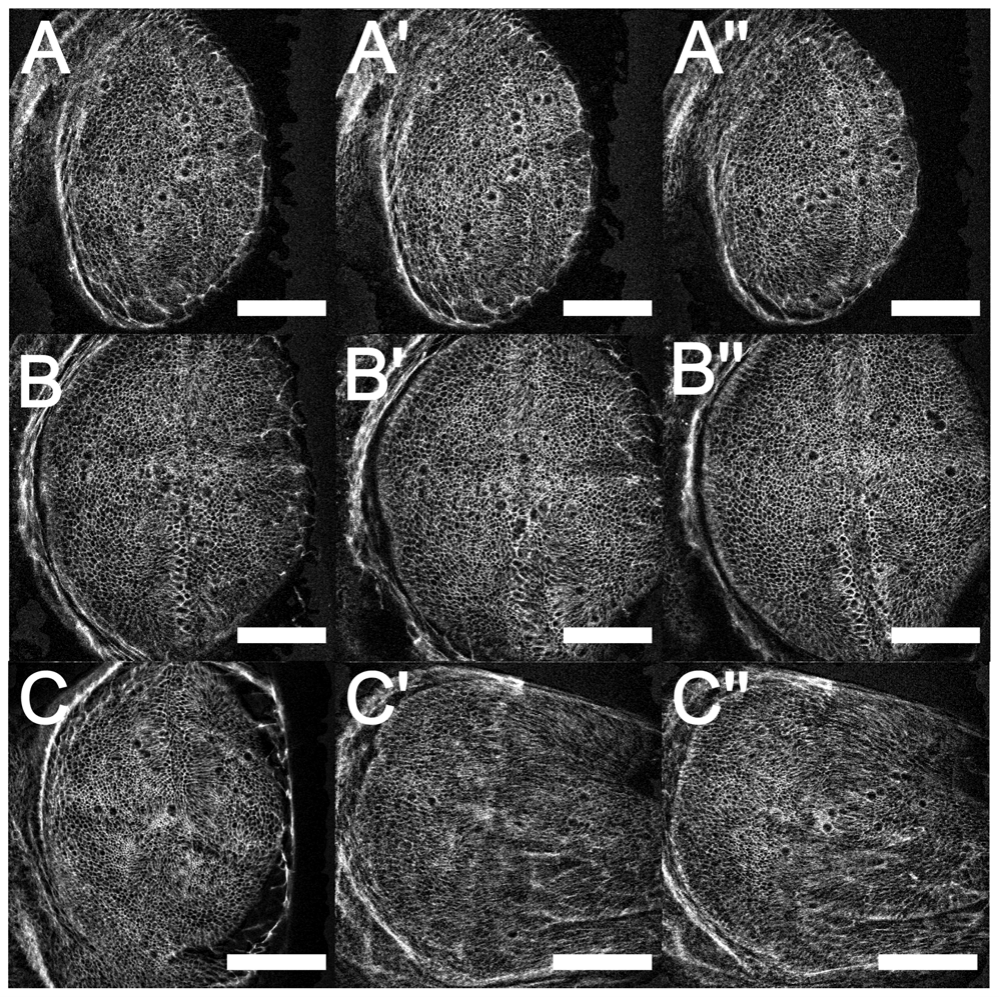
Images shown on the left (A,B,C) show the initial states of three different wing discs before stretching. The mitotic cells can be identified by their size and round shape. The number of mitotic cells in all these discs is comparable, as is expected for discs of the same age. The images in the middle (A′,B′,C′) show the discs directly after stretching, where A′ has not been stretched, B′ has been stretched with a force of 160 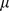
N and C′ has been stretched with a force of 350 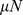. The images on the right (A″, B″, C″) show the same discs after 1 hour of stretching. The number of mitotic cells is higher in the stretched discs compared to the unstretched one. A quantitative comparison is given in Fig. 3. The discs are arranged such that the wing pouch is visible, with the dorsal side to the right. The scale bar corresponds to 50 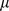
m.

To determine the cell proliferation rate while stretching, we label the cell junctions using a fluorescent marker (Lac-YFP [23]). As can be seen in the projections of confocal stacks in Fig. 2, this allows the determination of the number of cells in the field of view, as well as a quantification of the dividing cells. These can easily be identified since they increase their apical area by more than fivefold relative to the surrounding cells during division. In the following analysis, dividing cells are identified directly from the full three dimensional information of a confocal stack, rather than from a projection as shown in Fig. 2, to reduce uncertainty in the identification due to curvature of the tissue. In this way, the uncertainty in the number of dividing cells in a disc is less than 5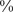, which is smaller than the variation observed between different discs. Since we are using a live-marker to observe cell shapes as a function of time, we can determine the number of cells added over a given time period to high accuracy irrespective of the time taken for mitosis. The cell proliferation rate is then given by [7]:
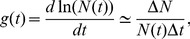
(1)where 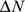 is the number of cells which divide during the time period 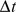. Here, we have used the approximation 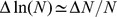, which is valid as long as the number of additional cells during a time period 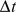 is small compared to the number of cells 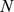 initially present.

In an experiment a given force is applied to the tissue and the number of additional cells is monitored from the newly appearing mitotic cells during the time-course of an hour under constant loading. Due to the fact that the number of cells in the field of view (see Fig. 2) is between 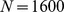 and 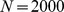, and the number of newly added cells ranges between 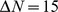 and 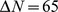, the approximation in Eq. 1 is valid. In order to be able to count all of the newly dividing cells, the temporal resolution of the imaging needs to be better than the time cells take to undergo mitosis. Previous determinations [24] have found a time for mitosis of 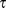 = 20 min to 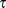 = 30 min depending on the temperature. During an experiment we thus image the cell outlines every 6 min, which allows an identification of the newly dividing cells even in the case of a strongly increased rate.

In Fig. 2, the cell outlines of three different wing discs are shown directly before (A, B, C) and after (A′, B′, C′) stretching as well as after 60 minutes of applying a constant force (A″, B″, C″). Disc A is left mechanically inert while discs B and C are stretched with different respective tensions. Apart from the mechanical stretching, the discs are treated identically with respect to dissection, culturing medium and attachment to the forcing apparatus. As can be seen by comparing Figs. 2A′, B′ and C′ with Figs. 2A″, B″ and C″, respectively, the mechanically stretched discs show an increase in the number of dividing cells relative to the unstretched one. All the discs were dissected from late third instar larvae, corresponding to the end of the growth period. Given a determination of the proliferation rate in-vivo at this stage of 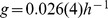 (see below), one expects 20(4) mitotic cells in the initial images (A,B,C), which is consistent with the observed number.

**Figure 3:**
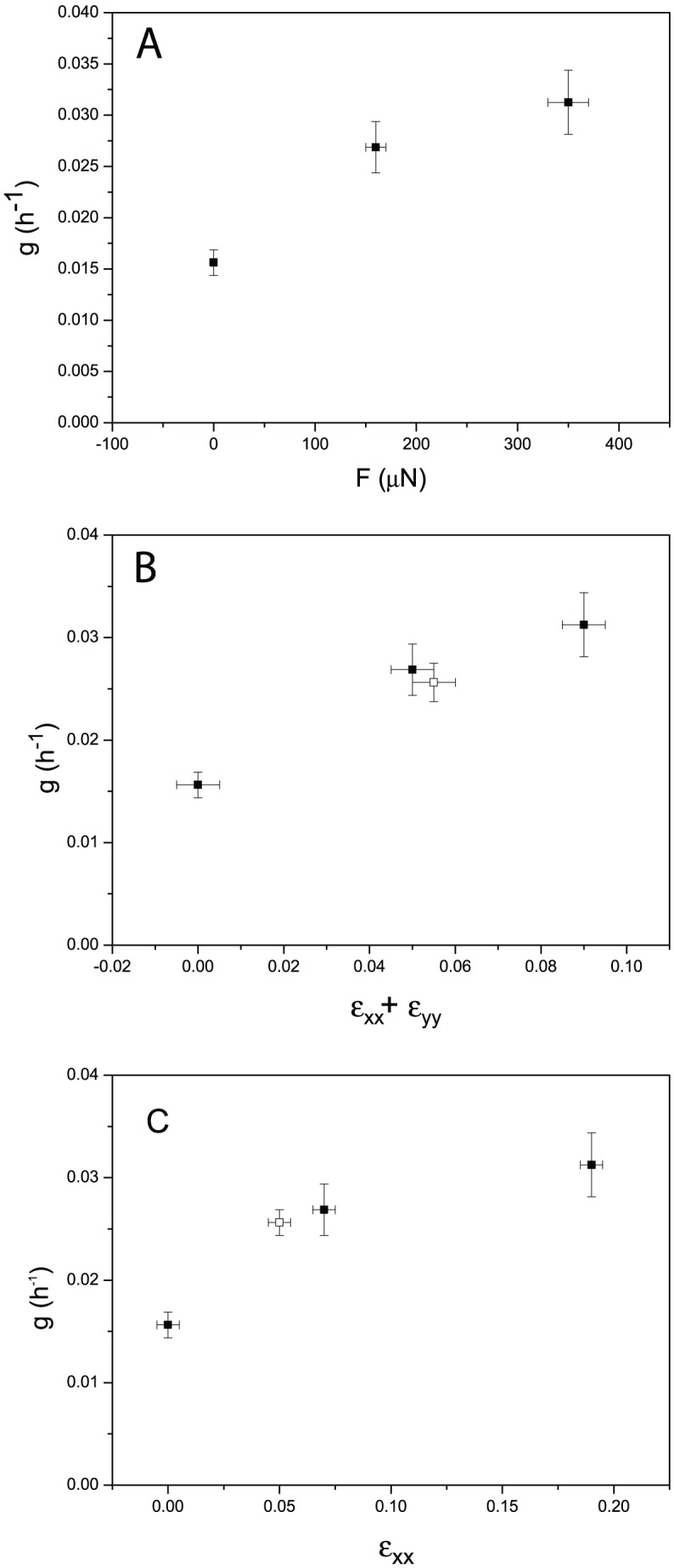
Force Dependence of the proliferation rate. A: In-vitro data for the force dependence of proliferation rates are shown for two different applied forces as well as the unperturbed control. Each data point shown corresponds to an average of at least five and up to eight different wing imaginal discs. B shows the same data, where the mechanical state is characterized by the trace of the strain 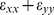. Also shown as an open symbol is the average of a set of experiments where wing discs buckled leading to a different tensional loading of the tissue, characterized by a lack of perpendicular strain. C again shows the same data versus the strain in the 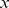-direction 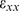 only. In this case the buckled tissue shows a different strain as the intermediate stretching, while showing the same proliferation rate.

The results of such experiments averaged over at least five wing discs subjected to the same force are shown in Fig. 3A. As can be seen, the proliferation rate in the unstretched discs is somewhat lower than that found in-vivo indicating that the culturing conditions, including the attachment to the cover slip, may not be ideal. However, under identical culturing conditions, yet in the presence of mechanical tension, the proliferation rate exceeds the in-vivo value in the late third instar, showing a twofold increase compared to the inert disc. No wounding of the tissue could be observed, although stronger forces of stretching than those studied here can lead to tearing and wounding of the tissue, which in turn might lead to a higher proliferation rate due to wound healing. The fact that the strain corresponding to the maximal tension applied is of the same order as the maximum strain found physiologically in-vivo during the early stages of growth (late 2nd instar) [22], also suggests that the increase in proliferation rate on mechanical pulling is unlikely to be a wounding response.

In order to directly test the effect of compressional forces on the tissue, we also applied such forces. Given the nature of the forcing setup, this however leads to a buckling, such that the previously flat tissue is bent in the 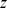 direction. Thus the apical side of the tissue is stretched in the process. However the stretching in this case is different from that arrived at by a tensional force in that there is no strain perpendicular to the applied force for the buckled tissue. This allows us to address the question of which type of strain is important for the mechanical feedback. A compressional strain, which occurs in the perpendicular direction in a stretching experiment, leads to a reduction of the proliferation rate in the mechanical feedback models. Since the models assume linear elasticity of the material, stress and strain are equivalent and the models actually calculate strains to determine the forces acting. In a buckling experiment only a tensional strain is acting. Thus given the same strain in the 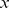 direction, different proliferation rates should be obtained in a stretching and a buckling experiment. This is shown in Fig. 3B and C, where the proliferation rate is plotted as a function of the trace of the strain 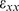 in part B and 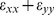 in part C. We see that the buckling experiment, shown by an open symbol agrees well with the stretching experiments (closed symbols) in the case of the total strain. This indicates that it is the trace of the strain, which controls the proliferation rate.

**Figure 4:**
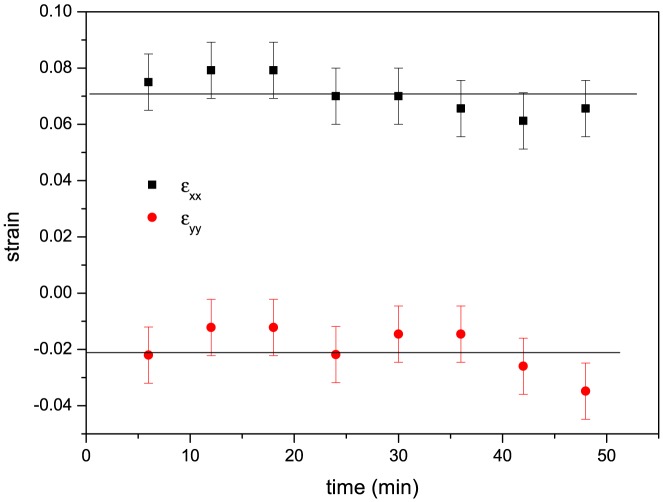
The mechanical strain of the tissue during stretching (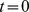 when stretching begins). Shown are the strain in the direction of the applied force (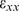) and in the perpendicular direction (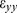).

For a time course of the strain in the tissue, the strain between consecutive images needs to be integrated over time. This is shown in Fig. 4 for one experiment, where a force of 160 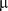
N was applied to the tissue. As can be seen from the figure, the strain remains constant within the errors, indicating the absence of creep in the tissue. The variation of the strains for different cell pairs observed in different regions of the wing disc (standard error of the mean ranging between 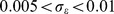) is comparable to the systematic uncertainty of the position determination for the centers of the cells, which is one to two pixels and corresponds to an error ranging between 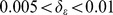. This is compatible with the fact that in the field of view the tissue is homogeneously stretched. Apart from the constant strain measured during the stretching, the absence of creep and thus plastic deformation can also be inferred from the elasticity of the response of the tissue after the force has been released, as has been shown in [21].

### B. In-vivo imaging

As mentioned above, while suitable for the time course of 1 hour, the culturing conditions used here are not ideal, such that the health of the cultured discs deteriorates over the course of several hours. Furthermore, due to handling issues the stretching method can currently only be applied to the relatively large late third instar discs. This means that the increase in the proliferation rate we find can only be demonstrated for wing discs during a short period at the end of their development. The mechanical feedback models however posit the regulatory nature of mechanical tension during the whole growth period. In order to assess this, we use in-vivo imaging over the entire period of development covering five days [22] to study the proliferation rate and mechanical strains at earlier times.

**Figure 5:**
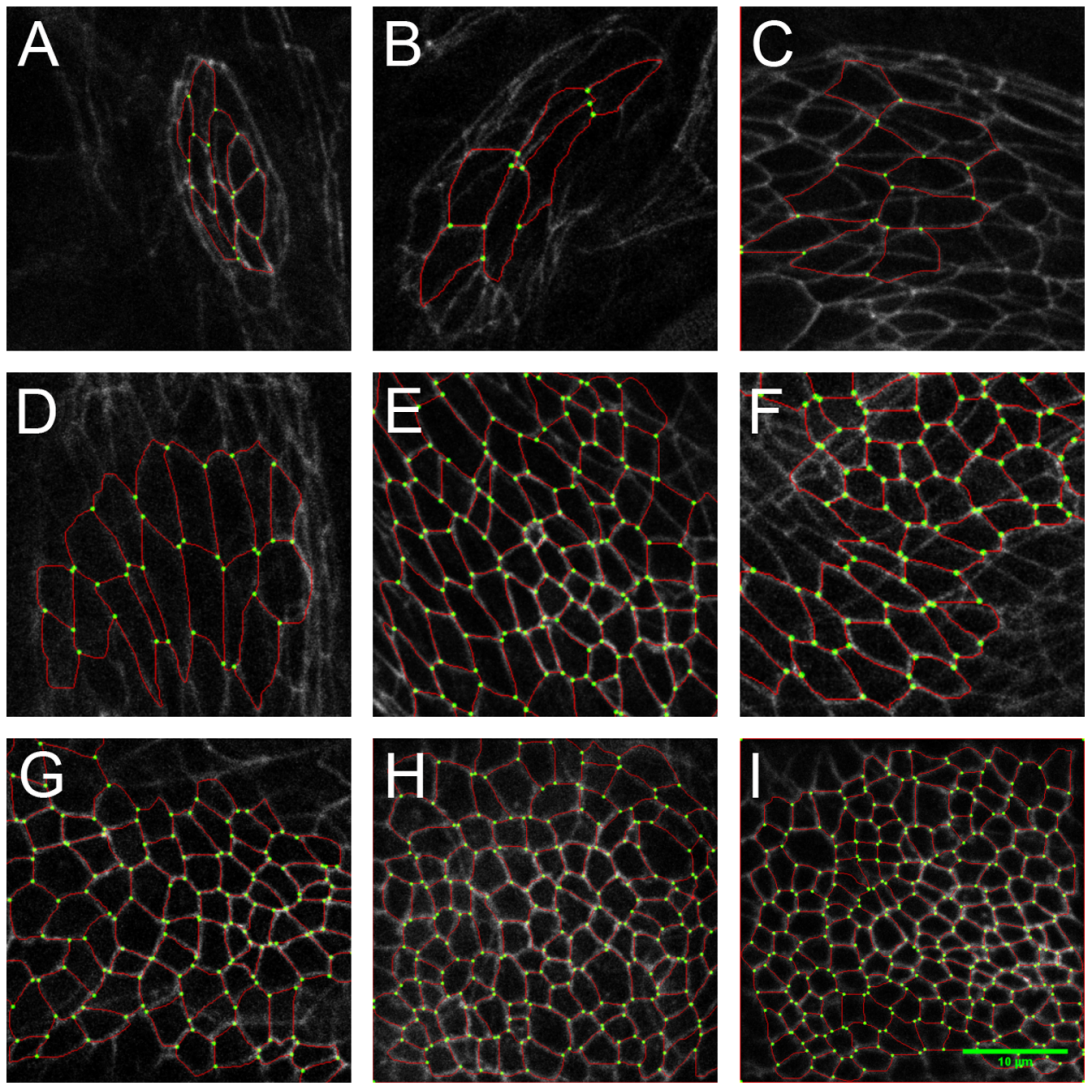
Cell junction outlines for the same wing disc as a function of time for nine different time points covering the whole of the developmental period spanning five days. The images correspond to time points of: A: 24 h, first instar; B: 32 h, second instar; C: 48 h; D: 56 h; E: 72 h; F: 80 h, third instar; G: 96 h; H: 104 h; and I: 120 h. The scale bar denotes 10 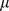
m. Identified cell outlines are superimposed in red. Note that in images of young discs, cells of the peripodial layer can be visible, which have not been identified in the segmentation.

**Figure 6:**
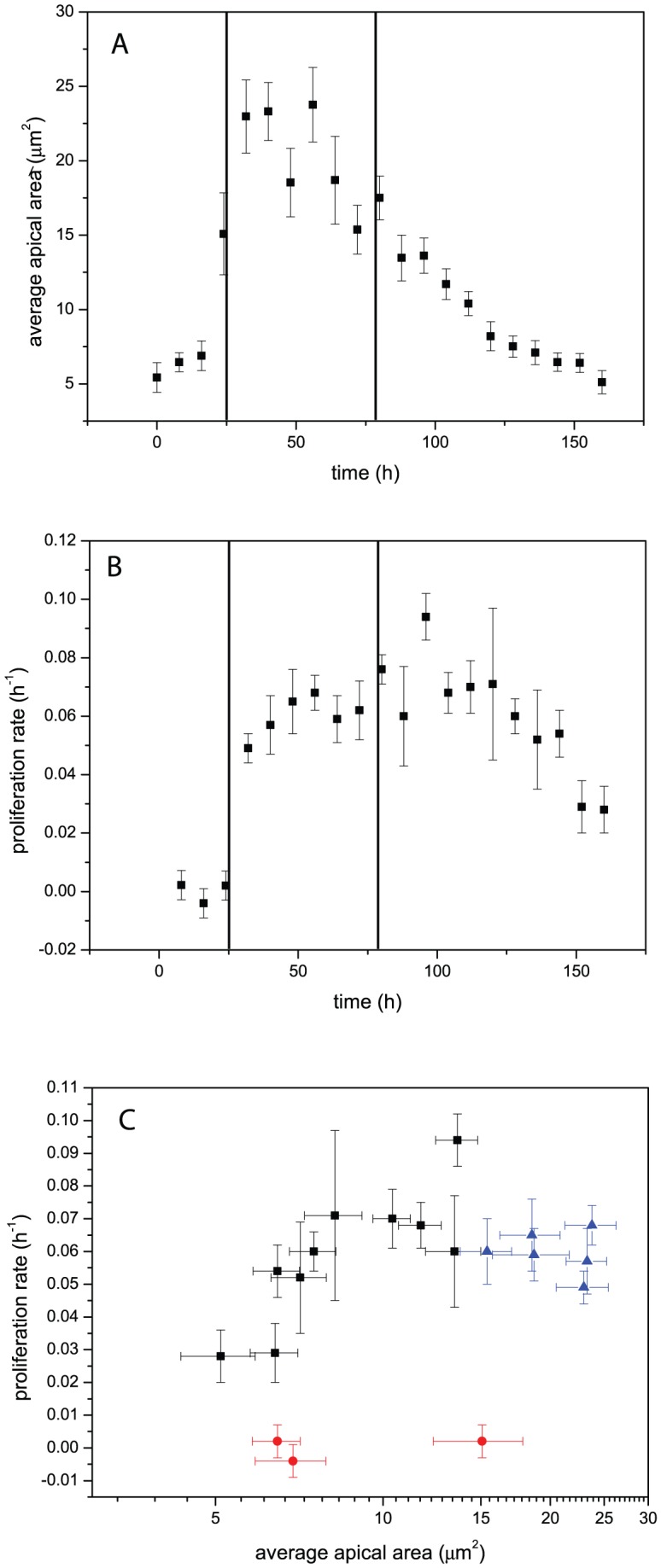
In-vivo imaging results. A: Dependence of the apical cell area on developmental time. B: Proliferation rate as a function of developmental time. The vertical lines indicate the time of the molts between different larval instars. Note the similar time evolution of the area and proliferation rate roughly compatible with a correlation between the two. The correlation is shown in C. Colors correspond to different instar stages (red first instar, blue second instar and black third instar), showing a significant correlation during the growth phase (i.e. in the late second and third instar).

For this purpose, we image cell outlines marked at the apical cell junctions using an E-Cadherin-GFP fusion protein [25] at several time points. Cell areas were determined using ImageJ and Packing Analyzer [26]. First, the StackReg plugin for ImageJ [27] was used to remove residual movement of the imaged larva from the stack showing the fluorescently labeled cell outlines. A 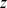-projection was then performed on the stack, resulting in an image of all the cell outlines in the field of view. This image was imported into Packing Analyzer where the cell outlines were determined manually and the cell areas calculated. The identified cell outlines are superimposed in Fig. 5. Next, the cell areas were averaged for each time step and larva. In order to be able to compare cell areas taken at different magnifications, all areas were determined in absolute units of 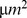. In this way, the development of the average cell area over time was determined for each larva. To account for age differences in the larvae at the onset of imaging, a time of 80 h was chosen as the beginning of the third instar and the times shifted up or down accordingly by time steps of 8 h. Cell areas were then averaged over all larvae for each time point (Fig. 6A).

The number of cells per disc 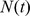 was determined for each larva and time point by dividing the respective disc area by the respective average cell area of the representative region. The division rate for each time point, 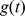, was then calculated via:
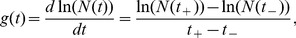
(2)where 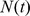 is the number of cells at time 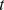. The second term shows the calculation of the average proliferation rate for discrete time steps, corresponding to 8 hours in between images in the experiments. 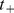 and 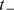 are the time-points after and before the time-point 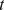, respectively. Here, the approximation of Eq. 1 can no longer be made, since the number of additional cells is comparable to and may even exceed the number of initial cells during the time course of 8 hours. Finally, these division rates were filled into a table identical to the table used to calculate the average cell areas and the average division rate for each time point was calculated (Fig. 6B).

Given these two measures (proliferation rate and apical area), we have determined the time dependence of proliferation on the one hand and a correlator of mechanical stress on the other hand. In Fig. 6C, we see a correlation between the apical area and the proliferation rate during the entire third instar stage, which can be quantified with a Spearman's rank of 0.8, corresponding to a p-value of 0.01. This is similar to what has been found in cell cultures [28]. Since there is no mechanical access inside the larva, these measurements are necessarily correlative. Given the inverse correlation of compressional stress to apical area [15] this implies a negative correlation between mechanical compression and proliferation rate. Therefore, the effect of mechanical forces we show by direct manipulation in late third instar wing discs appears to be present during the whole growth period. An indication of the causative nature of this connection can be inferred from the fact that considerable forces are exerted on the wing disc by a muscle fiber [22], [29] at the time-point in development where proliferation is highest.

## Conclusions

According to mechanical force models for size regulation, mechanical compression increases in the center of the disc over time [11]–[13] and is involved in growth termination. The most important prediction of these models, the formation of a mechanical stress gradient, has been confirmed previously [13]–[15]. This paper provides evidence in favor of the most important assumption underlying these models, namely the regulation of growth by mechanical forces, where we have shown that the proliferation rate increases on mechanical stretching. A direct demonstration of a decrease in proliferation on compression remains a technical challenge. These observations taken together show that mechanical forces are a key regulator of growth and important in the control of organ size. Since mechanical forces have also been shown to regulate growth in mammalian cells, it would be interesting to study whether similar organ size regulation mechanisms are involved in mammalian tissues as well. Several key players have been identified in this mechanical regulation of growth [19], which also have a role in the wing imaginal disc, but the connection to the known signaling pathways remains to be elucidated.

## Materials and Methods

The following stocks were used for the experiments described:

E-Cad-GFP-III [25]

w; Lac-YFP (CPTI-002601)/(SM6a) [23]

### In-vivo Imaging

In-vivo imaging is performed as described in [22]. Larvae are placed between a microscope slide and a cover slip (thickness 170 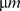). The size of the cover slip used is adjusted for the age of the larva. Excess water is removed, thus immobilizing the larva by surface tension and the pressure imposed by the cover slip. Using an upright Leica SP1 confocal microscope, the position of the larva between the two glass slides is adjusted such that the wing imaginal disc is in clear view. The SP1 is then used in the fluorescence confocal mode to obtain stacks of the GFP marked cell outlines of the ECad-GFP flies. Images are taken at reduced laser power (Ar ion laser at 30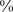 power setting corresponding to roughly 0.1 mW) as a precaution to prevent phototoxicity.

### Image Analysis

Images are processed using ImageJ. Image analysis of the stacks obtained as described above is mostly needed to correct for residual small movements of the larvae between single slices of a stack. The StackReg and TurboReg plugins [27] were used for this.

In practice, the image slice out of a stack, which best shows the cell outlines, is first chosen. Slices showing no fluorescent signal are deleted, as are slices showing the peripodial membrane. For early stage discs, where the peripodial membrane has the same features as the columnar layer, the layers are separated by their z-position for cell segmentation. Slices which were taken while the larva moved excessively (i.e. more than a cell diameter) are also removed. The fluorescence images are then aligned with respect to the chosen image using the aforementioned plugins. Finally, an average z-projection of the fluorescent channel is taken.

The z-projected cell outlines are then analyzed further using Packing Analyzer, where the cell outlines are identified. This identification is curated manually for all cells. After segmentation of the cells, the apical areas are approximated as polygons defined by the positions of the cell vertices, i.e. the point where three cells meet. From this approximation, their respective (apical) areas are determined. This is done for all the cell outlines in the field of view, thus giving a value for the average apical cell area.

To calculate the division rate, the area of the entire wing disc was first determined. Again, the StackReg plugin [27] was used to remove larval movement and a z-projection of the stack showing the whole disc performed. The ‘Polygon selections’ tool in ImageJ was then used to outline the fluorescently labeled area. This area was subsequently measured using ImageJ and normalized in the same way as the cell areas.

### Mechanical Perturbation

Wing imaginal discs are dissected in the late third instar stage in isotonic PBS solution. Dissected discs expressing Lac-YFP markers are then attached with the basal ventral end to a glass slide coated in poly-lysine solution via electrostatic interactions. The corresponding dorsal end of the disc is attached to a moveable cover slip also coated in poly-lysine solution. The moveable cover slip is in turn attached to a spring sheet, which is used to apply a calibrated force to the wing disc tissue as discussed in [21] (see also Fig. 1C). After the disc has been attached, the PBS solution is removed by suction and Clone8 culture medium is applied to the disc. The culturing medium is exchanged every 30 minutes. After 60 minutes of stretching the discs can still be induced to evaginate upon treatment with ecdysone.

In order to be able to determine the cell outlines while stretching the tissue, the setup was custom built on an inverted confocal microscope (Leica SP1). The overall mechanical response of the tissue is recorded in transmission mode at a magnification of 10×. For the determination of cell outlines, a magnification of 40× is used in the fluorescent confocal mode. The illumination laser is a CNI-LPSS solid state laser with a wavelength of 473 nm used at a power of 0.1 mW at the sample to avoid bleaching and phototoxicity.

Once the tissue is stretched, stacks are taken every 6 minutes and analyzed in ImageJ. For the determination of dividing cells, the stack is analyzed in three dimensions meaning that mitotic cells are identified in individual layers of the stack. This is necessary due to the curvature of the disc tissue. Individual mitotic cells are followed in time and newly dividing cells as well as cells which have finished dividing are thus identified at each time point. In the determination of the cell division rate as discussed in the main text, the newly dividing cells thus identified are counted over the time course of 1 hour to give 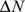 in Eq. 1.

The forces acting on the tissue are determined both from the applied force given by the spring sheet as well as from the previously determined elastic properties of the wing disc and the mechanical strain determined from the transmission image [21]. For the strain determination, the distance between fixed points in the disc before and after stretching is determined. As fixed points we use mitotic cells, which can be easily identified and which are apart far enough such that the change in position can be determined to sufficient accuracy. The distances between two such points is taken in the 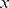 as well as in the 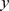 direction, such that we can determine 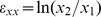, as well as 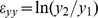. Here, 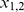 are the distances between the fixed points in the 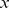 direction before and after stretching respectively. The same applies for 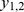 for the 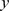 direction. The true strain is used here as discussed in the main text. Note that for small differences between the lengths, the true strain is exactly equal to the usual definition of strain, i.e. 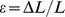.
